# Interleukin-33 (IL-33) as a Diagnostic and Prognostic Factor in Traumatic Brain Injury

**DOI:** 10.1155/2020/1832345

**Published:** 2020-01-10

**Authors:** Ali Kemal Erenler, Ahmet Baydin

**Affiliations:** ^1^Hitit University, Faculty of Medicine, Department of Emergency Medicine, Çorum, Turkey; ^2^Ondokuzmayıs University, Faculty of Medicine, Department of Emergency Medicine, Samsun, Turkey

## Abstract

Interleukin-33 (IL-33) is a cytokine involved in interleukin-1 family. Role of IL-33 in immune system activation is well described in the literature. IL-33 has been identified as an endogenous alarm signal (alarmin) to alert various types of immune cells to trauma. In this narrative review, we aimed to underline the diagnostic and prognostic importance of IL-33 in trauma, particularly in brain trauma.

## 1. Introduction

IL-33 is known to be released from damaged cells following trauma. It is also known as an “alarmin” which means an endogenous signal of tissue injury [1]. It is a nuclear-associated cytokine belonging to IL-1 family and well known with the inducer role in type-2 allergic immunity [2]. In central nervous system (CNS), IL-33 activates microglias and macrophages in order to limit glial scarring [3, 4]. Here, we sought the utility of IL-33 in traumatic brain injury (TBI).

## 2. Materials and Methods

This narrative review was conducted by entering keywords “interleukin-33” or “IL-33” and “trauma” into MEDLINE/PubMed, EMBASE, and CINAHL scientific databases. During the database search, we excluded studies which did not relate with the objective by reading the title and abstract. Studies that were not published in English, studies without explanatory abstracts, and studies that do not focus on brain injury were excluded. Two reviewers conducted independent screening and data extraction. First, the reviewers independently screened titles and abstracts of the returned articles to decide if they met the inclusion criteria. Original articles and reviews published in the last 5 years were preferred. If there were studies from the same source, the more recent or more informative study was selected. A total of 23 articles out of 71 studies were included into study.

## 3. Structure and Function of IL-33

Alarmins, including IL-33, are released when a trauma occurs, and their role is to activate immune system against damage [5]. IL-33 is a member of the IL-1 cytokine family along with IL-1*α*, IL-1*β*, and IL-18 and expressed in structural and lining cells exposed to the environment including fibroblasts, endothelial, and epithelial cells of skin, gastrointestinal tract, and lungs [6, 7].

IL-33 shows its effects by activating signaling pathways depending on gene 88 (MyD88) of immune cells which is the primary response gene on the myeloid differentiation expressing the cytokine receptor IL-1 receptor-like 1 (ST2) molecule and signals through a heterodimeric receptor complex comprising an IL-33-specific ST2 coupled with the coreceptor IL-1 receptor accessory protein (IL-1 RAcP), which belongs to other members of the IL-1 cytokine family [8–10]. Following proteolytic cleavage of its precursor pIL-33 (full-length IL-33), IL-33 is considered to be released to the extracellular space. When exogenous pathogen-associated molecular patterns (PAMPs) are triggered, IL-33 is actively released; however, there is lack of evidence that it is secreted from dendritic cells or mast cells. Unlike other members of the IL-1 family, active IL-33 secretion is independent of caspase-1 and caspase-8 (required for cleavage of pIL-1*β* and/or pIL-18) or calpain (required for cleavage of pIL-1*α*). In vitro, recombinant pIL-33 is cleaved by recombinant caspase-1; however, role of caspase-1 in the cleavage of pIL-33 is not clear [11].

IL-33 serves as an inhibitor of autophagy, and to our knowledge, it is expressed in various organ tissues in the human body, such as endothelial, bronchial, and intestinal epithelial cells [12, 13]. It is also known that IL-33 is mainly secreted from macroglia, including grey-matter astrocytes and oligodendrocytes [2].

## 4. IL-33 and Traumatic Brain Injury

It is well documented in the literature that TBI is a major cause of morbidity and mortality worldwide. The damage mechanism of TBI is a complex process that may mainly be divided into two categories as primary and secondary brain injury. Primary brain injury is a type of injury directly caused by external force, and the secondary brain injury is a sort of indirect injury containing a complex process in which inflammation plays an essential role [14].

Following an injury or an organ damage and regardless of its type (physical, chemical, or metabolic assault), cells die by necrosis. Following necrosis, rupture and disintegration in a sterile environment occur. This type of traumatic cell death also results in the accumulation of inflammatory cells to the injury site, and a massive immune response triggered by recognition of such danger or alarm signals emerges [15]. Biomarkers such as IL-33 have been considered in the early hours after TBI, and it has been suggested that they could guide clinicians in planning the next stages of patient management [16–19] on a more general perspective, and it should be noted that in some instances, biomarkers already hold the potential for prognostication of mid- and long-term outcomes [20, 21]. The importance of biomarkers is also meant to grow because of innovation in biomedical engineering and point of care detection, with the ultimate goal of coupling them with neuroradiological patterns to define robust biosignatures for TBI patients [22].

Proinflammatory cytokines of the IL-1 family (IL-1*α*, IL-1*β*, IL-18, IL-33, and IL-36) play essential role on inflammatory and immune processes. These cytokines are released during the early stages of inflammation and, as mentioned before, have been named “alarmins” because they alert the host to induce an inflammatory reaction [12].

In a report, it was stated that the combination of markers including IL-33 improved the prognostic performance and might be used as a useful tool for risk determination in trauma patients [23].

A study with mice showed that IL-33 appeared to downregulate the autophagic activation of apoptosis and the inflammatory response. This downregulation results in protection of mice against injury from collagenase-induced intracerebral hemorrhage. Also, inhibitor role of IL-33 in autophagic activity and apoptosis in neonatal rats was reported. Thus, neurons were not affected from recurrent seizures [12].

In an animal study by Heuvel et al., a weight-drop TBI model was performed to mice, and inflammatory cytokines were measured. In the study, the serum levels of IL-33, IL-1*β*, IL-38, TNF-*α*, IFN-*α*, and IL-19 in the hippocampus were found to be elevated at 3-hour time point [24].

The relationship between trauma and IL-33 was reported in a study with 472 patients. It was found that plasma IL-33 levels elevated on admission and over time in a positive correlation with other cytokines IL-4, IL-5, and IL-13 in patients with blunt trauma [25].

In another study, it was shown that mortality in hospital following blunt trauma was associated with increased plasma sST2 concentrations and suppressed IL-33 levels. Additionally, sST2 levels were found to correlate with injury severity, organ dysfunction, and altered inflammatory response. According to these results, it was concluded that the ST2/IL-33 axis played an important role in the human response to trauma. It was also concluded that sST2 might be a useful biomarker in trauma severity prediction [26].

Elevated serum IL-33 levels after multiple injuries indicate that IL-33 can be referred as an alarmin in human response to polytrauma. It may also be an indicator of the amount of damaged structural cells by mechanical effects of trauma [27]. Damage to central nervous system (CNS) results in IL-33 release, particularly from oligodendrocytes and astrocytes [28].

Foster et al. hypothesized that secretion of IL-33 in response to CNS trauma might initiate a protective feedback loop on neurons involving T cells, glial cells, and monocytes [1]. It was also reported that meningeal ILC2s were activated in an IL-33-dependent manner after CNS injury [29].

In a study using human TBI microdialysate, tissue sections from human TBI, and mouse models of CNS injury, it was revealed that expression of IL-33 in the brain was elevated reaching a maximum after 72  hours in both human samples and mouse models. It was also reported that IL-33 was mainly produced by astrocytes and oligodendrocytes. When brains of mice were deficient in ST2, IL-33 receptor, number of microglia, and macrophages were decreased, and in response damage, their cytokine and chemokine status was altered. These results indicated the essential role of IL-33 in neuroinflammation following TBI. Moreover, IL-33 targets microglia and macrophages in response to damage [30]. Similarly, spinal cord injuries (SCIs) also result in increased IL-33 levels. In the subchronic SCI stage, IL-33 remains elevated particularly in astrocytes' nuclei. Pomeshchik et al. demonstrated that when recombinant IL-33 was given after contusion, SCI results in improved and long-lasting motor recovery, and secondary tissue damage reduces. Even following the initial dose of IL-33, the expression of cytotoxic TNF-*α* in the injured spinal cord reduced [4]. Literature involved in this review on usefulness of IL-33 in TBI is summarized in Table 1.

## 5. Conclusion

ST2/IL-33 cytokine signaling system has emerged as an intercellular signaling system that participates in processes of the immune response, homeostasis, and tissue injury/repair. IL-33 is released by endothelial cells, immune cells, and epithelial cells as a result of cell injury or death. There are many extracellular actions of IL-33 including type-2 cytokine production, epithelial repair, and regeneration through amphiregulin, activation of ST2-positive Tregs, and polymorphonuclear neutrophil recruitment and activation [26]. It may be concluded that IL-33 levels tend to increase following TBI. Thus, in combination with other cytokines that take part in tissue damage, it may be used as a diagnostic and prognostic indicator of TBI severity. However, as an inflammatory marker, it must be kept in mind that IL-33 may elevate in various conditions, and diagnosis of TBI must not be based solely on serum IL-33 level.

## Figures and Tables

**Table 1:** Comparison of studies on IL-33 in traumatic brain injury involved in the review.

Study type	Study design	Conclusion	Reference
Original article	A blunt, weight-drop approach to model TBI in mice.	It was shown that TBI causes the elevation of IL-33, IL-1*β*, IL-38, TNF-*α*, IFN-*α*, and IL-19 in the hippocampus at 3 h time point, and concomitant EI results in the dose-dependent downregulation of IL-33, IL-1*β*, IL-38, TNF-*α*, IFN-*α*, and IL-19.	[24]

Original article	Spinal cord of mice was damaged, and recombinant IL-33 was injected.	Addition of wild-type lung-derived ILC2s into the meningeal space of IL-33r-/- animals partially improves recovery after spinal cord injury. IL-33 released after CNS injury not only initiates a local response but also a meningeal one through actions of ILC2s.	[29]

Comment	Comment on “The glia-derived alarmin IL-33 orchestrates the immune response and promotes recovery following CNS injury” by Gadani SP, Walsh, J. T., Smirnov, I., Zheng, J. and Kipnis, J. published in neuron in 2015.	Administration of recombinant IL-33 might be beneficial for treating TBI.	[1]

Original article	Transient focal ischemia was induced by intraluminal occlusion of the left middle cerebral artery for 1 h with silicone-coated sutures in mice.	IL-33/ST2 signaling was described as a potential immune regulatory mechanism that enhances the expression of IL-10 in M2 microglia and reduces acute ischemic brain injury after stroke.	[28]

Original article	Serum ST2 concentrations in 106 healthy controls and 106 severe TBI patients were measured.	Serum ST2 concentrations are significantly related to inflammation. In TBI, it may be a potential diagnostic marker.	[14]

Original article	Samples from human TBI microdialysate, tissue sections from human TBI, and mouse models of CNS injury were used.	IL-33 plays a role in neuroinflammation, and microglia/macrophages are cellular targets for this IL following TBI.	[30]
